# Amendments to the Medical Practice Law

**Published:** 1895-06

**Authors:** 


### AMENDMENTS TO THE MEDICAL PRACTICE LAW.

THE following amendments to the medical practice law of 1893 were passed by the last legislature and filed in the office of the secretary of state, May 13, 1895.

I. Providing penalties for violation of the medical practice law:

An act to amend chapter 661 of the laws of 1893, entitled "An act in relation to the public health, constituting chapter twenty-five of the general laws."

*The People of the State of New York, represented in Senate and Assembly, do enact as follows:*

SECTION 1. Section 153 of chapter 661 of the laws of 1893, entitled "An act in relation to the public health, constituting chapter twenty-five of the general laws," is amended so as to read as follows:

SEC. 153. Penalties and their collection.—Any person who, not being then lawfully authorized to practise medicine within this state and so registered according to law, shall practise medicine within this state without lawful registration or in violation of any provision of this article; and any person who shall buy, sell or fraudulently obtain any medical diploma, license, record or registration, or who shall aid or abet such buying, selling or fraudulently obtaining, or who shall practise medicine under cover of any medical diploma, license, record or registration illegally obtained, or signed, or issued unlawfully or under fraudulent representations or mistake of fact in a material regard, or who, after conviction of a felony, shall attempt to practise medicine, or shall so practise, and any person who shall append the letters M. D. to his or her name, or shall assume or advertise the title of doctor (or any title which shall show or tend to show that the person assuming or advertising the same is a practitioner of any of the branches of medicine) in such a manner as to convey the impression that he or she is a legal practitioner of medicine or of any of its branches without having legally received the medical degree, or without having received a license which constituted at the time an authority to practise medicine under the laws of this state then in force, shall be guilty of a misdemeanor, and on conviction thereof shall be punished by a fine of not more than $250, or imprisonment for six months for the first offense, and on conviction of any subsequent offense, by a fine of not more than $500 or imprisonment for not less than one year, or by both fine and imprisonment. Any person who shall practise medicine under a false or assumed name, or who shall falsely personate another practitioner of a like or different name, shall be guilty of a felony. When any prosecution under this article is made on the complaint of any incorporated medical society of the state, or any county medical society of such county entitled to representation in a state society, the fines when collected shall be paid to the society making the complaint, and any excess of the amount of fines so paid over the expense incurred by the said society in enforcing the medical laws of this state, shall be paid at the end of the year to the county treasurer.

SEC. 2. This act shall take effect immediately.

II. Providing for better general education preliminary to receiving the degree of bachelor or doctor of medicine:

An act to amend chapter 661 of the laws of 1893, entitled "An act in relation to the public health, constituting chapter twenty-five of the general laws."

*The People of the State of New York, represented in Senate and Assembly, do enact as follows:*

SECTION. 1. Section 145 of chapter 661 of the laws of 1893, entitled "An act in relation to the public health, constituting chapter twenty-five of the general laws," is amended so as to read as follows:

SEC. 145. Admission to examination.--The regents shall admit to examination any candidate who pays a fee of $25 and submits satisfactory evidence, verified by oath if required, that he: (1) Is more than twenty-one years of age; (2) is of good moral character; (3) has the general education required in all cases after August 1, 1895, preliminary to receiving the degree of bachelor or doctor of medicine in this state; (4) has studied medicine not less than three full years, including three satisfactory courses in three different academic years in a medical school registered as maintaining at the time a satisfactory standard; (5) has either received the degree of bachelor or doctor of medicine from some registered medical school or a diploma or license conferring full right to practise medicine in some foreign country. The degree of bachelor or doctor of medicine shall not be conferred in this state before the candidate has filed with the institution conferring it the certificate of the regents that three years before the date of the degree he has either graduated from a registered college or satisfactorily completed a full course in a registered academy or high school; or had a preliminary education considered and accepted by the regents as fully equivalent; or had passed regents' examinations representing for degrees conferred in 1898 one year of academic work, for degrees conferred in 1899 two years of academic work, and for degrees conferred in 1900 a full high-school course. Students who had matriculated in a New York medical school before June 5, 1890, shall be exempt from this preliminary education requirement, provided the degree be conferred before August 1, 1895. The regents may, in their discretion, accept as the equivalent for any part of the third and fourth requirement evidence of five or more years' reputable practice, provided that such substitution be specified in the license.

SEC. 2. This act shall take effect immediately.

---

MEDICAL LEGISLATION IN MONTANA.—A new Medical Practice Act, to go into effect July 1, 1895, has lately been passed by the Montana Legislature, and received the approval of the Governor on March 13th. The act provides for the appointment by the Governor of a board of seven medical examiners constituted of graduates of accredited colleges of medicine. Applicants for a license to practise, graduating after July 1, 1898, must have attended four courses of lectures of at least six months each. The board has the privilege both to refuse to grant and to revoke a certificate for unprofessional, dishonorable or immoral conduct.—*Medical News.*

---

### TRIONAL IN THE INSOMNIA OF CHILDREN.

IN A paper on this subject Claus concludes (*Theraupeutic Gazette*):

1. Trional, in the dose of one-third to twenty-two grains, according to the age of the child, is a brilliant hypnotic. On the following morning neither headache nor heaviness of the head was noticed. Physiological sleep was favored. Patients do not become accustomed to it. Sleep occurred in ten or fifteen minutes after its administration.

2. Trional has no very pronounced effect upon insomnia the result of pain.

3. Trional leaves the intellectual, respiratory and circulatory functions untouched, and it has a favorable effect upon digestion.

4. In toxic insomnia, particularly that caused by alcohol, chloral seems to be more active.—*Atlanta Med. and Surg. Journal.*

---

WHEN a physician in Arkansas becomes an habitual drunkard the state board of health is by law enjoined to revoke his license.

